# A middle-range model for improving quality of nursing education in Malawi

**DOI:** 10.4102/curationis.v41i1.1766

**Published:** 2018-03-22

**Authors:** Thokozani M. Bvumbwe, Ntombifikile G. Mtshali

**Affiliations:** 1 Faculty of Health Sciences, Mzuzu University, Malawi; 2 School of Nursing and Public Health, University of KwaZulu-Natal, South Africa

## Abstract

**Background:**

Despite a global consensus that nurses and midwives constitute the majority and are a backbone of any country’s health workforce system, productive capacity of training institutions remains low and still needs more guidance. This study aimed at developing a middle-range model to guide efforts in nursing education improvements.

**Objective:**

To explore challenges facing nursing education in Malawi and to describe efforts that are being put in place to improve nursing education and the process of development of a model to improve nursing education in Malawi.

**Method:**

The study used a qualitative descriptive design. A panel discussion with eight nursing education and practice experts was conducted guided by core concepts derived from an analysis of research report from a national nursing education conference. Two focus group discussions during two quarterly review meetings engaged nurse educators, practitioners and clinical preceptors to fill gaps from data obtained from a panel discussion. A qualitative abductive analysis approach was used for the development of the model.

**Results:**

Transforming and scaling up of nursing education emerged as the main concept of the model with nursing education context, academic practice partnership, regulation, competent graduate and nursing workforce as sub concepts. Key main strategies in the model included curriculum reforms, regulation, transformative learning, provision of infrastructure and resources and capacity building.

**Conclusion:**

The model can be used to prioritise nursing education intervention aimed at improving quality of nursing education in Malawi and other similar settings.

## Background

Malawi is among countries with the most strained health systems in sub-Saharan Africa characterised by a heavy burden of disease (Bandazi et al. 2013; Tumusiime et al. 2012). There is high prevalence of diseases such as tuberculosis, malaria, HIV and AIDS and other tropical diseases. Non-communicable diseases are also on the increase (Lupafya et al. 2016). Malawi still registers poor healthcare indicators such as maternal mortality ratio of 634 per 100 000 live births (World Health Organization 2015), infant mortality rate of 42 per 1000 live births and under five mortality rate of 64 per 1000 live births (National Statistical Office 2016). Severe shortage of health professionals significantly contributes to the poor health indicators (Bradley et al. 2015).

Health workforce is key to strengthening the health system for attainment of health-related Sustainable Development Goals (SDGs) (Senkubuge, Modisenyane & Bishaw 2014). There is a global consensus that nurses and midwives constitute the majority of the global health workforce (Global Health Workforce Alliance 2013). Considering that nurses form the universal access point for almost 90% of healthcare users in most sub-Saharan countries, including Malawi (Makaula et al. 2012; Manjomo et al. 2016), production of a competent and effective nursing workforce therefore is key to transform the nursing landscape. However, Malawi currently has 33% of the healthcare workers necessary to effectively deliver healthcare to the population (Bandazi et al. 2013) and still needs 20% more nurses to meet the country’s healthcare needs. The current productive capacity of training institutions is way below the required capacity to achieve such a goal (Middleton et al. 2014; Schell et al. 2011).

### Problem statement

The plan of action for scaling up of quality nursing and midwifery education and practice for the African region 2012–2022, transforming and scale-up of health professionals’ education and training guidelines, the regional professional regulatory framework for nursing and midwifery and the four-year integrated nursing and midwifery competency-based prototype curriculum for the African region provide adequate framework for scaling up of nurses and midwives at the regional level. However, Mtshali et al. (2007) reported that many countries across sub-Saharan Africa do not adhere to such guiding frameworks. The Lancet Commission and the Global Health Workforce Alliance affirms that professional education has generally not kept up with the pace of healthcare demands and challenges (Frenk et al. 2010; Global Health Workforce Alliance 2013). Many nursing programmes are still fragmented, outdated and lack teamwork in training of healthcare workers. Most of the programmes are still hospital oriented at the expense of primary healthcare.

Literature from sub-Saharan Africa highlights numerous challenges facing nursing education in the region, including lack of teaching and learning resources, unresponsive curriculum and poor collaboration between academia and practice setting (Middleton et al. 2014). The United States President’s Emergency Plan for AIDS Relief (PEPFAR) in response to key capacity building challenges facing nursing education in sub-Saharan Africa partnered with Malawi Government in 2011 through a Nursing Education Partnership Initiative (NEPI) to strengthen education systems, institutions and organisations as well as faculty capacity building (Middleton et al. 2014). This was in support for the implementation of the Malawi Health Sector Strategic Plan 2011–2016.

The Malawi National Health Strategic plan 2011–2016 plans to strengthen performance of the health system in order to support delivery of essential health package among other objectives. The strategic plan has prioritised capacity building of training institutions to achieve improved healthcare service delivery. A number of strategies have been implemented, such as nurse educators’ development, clinical training strengthening, review of cost-effective interventions to increase students’ intake, curriculum reviews, introduction of incentive packages for nurse educators, provision of teaching and learning material and implementation of an operational plan for increasing nursing intake (Government of Malawi 2011). However, experience shows that these efforts appear fragmented. It is against this background that the purpose of this study was to explore how the quality, quantity and relevance of nursing education are being improved in Malawi. Subsequently, the aim was to develop a middle-range model that would guide improvement in the quality, quantity and relevance of nursing education in the local context of Malawi.

## Purpose of the study

The purpose of this study was to describe a middle-range model developed to guide efforts to improve the quality, quantity and relevance of nursing education in the local context of Malawi.

## Research objectives

The specific objectives of this study were to:

- explore challenges facing nursing education in Malawi
- describe efforts that are being put in place to improve nursing education in Malawi
- describe the process of development of a model to improve nursing education in Malawi.

### Definitions of keywords

*Model* is a set of well-developed categories where concepts and sub-concepts are systematically interrelated through statements of relationships to explain a phenomenon (Corbin & Strauss 2014). Middle-range models are more concrete, narrow and limited in scope than grand theories.

*Nursing education* is a system that functions to prepare nursing graduates for practice.

*Quality* refers to the qualifications of health professionals and the adequacy of these qualifications to address the health needs of a specific population.

*Quantity* refers to the number of health professionals and the adequacy of that number to address the health needs of a specific population.

*Relevance* is the relevance of health professionals’ education to meet the current and future health needs of specific populations, including an appropriate skill mix and equitable distribution and availability of health professionals to the local context.

### Research methods and design

#### Design

The study used qualitative descriptive design, which helped to provide adequate information about the themes that were derived from the analysis of a national nursing education conference. During the model development stage, a qualitative abductive analysis approach was used (Timmermans & Tavory 2012). Abductive approach was well suited for this study because the researcher decided what proceeding steps to take based on the data that were collected. Model development was based on focus group interviews and the researcher’s experiential knowledge of nursing education in Malawi (Marion 2002).

#### Study population and sampling

The study population of this study comprised nursing educators, nurse practitioners and clinical preceptors and officers from nurses and midwives regulatory body. Firstly, eight participants were purposively sampled for a panel discussion based on their expertise in nursing education and nursing practice. Subsequently, participants from training institutions and clinical preceptors who attended NEPI quarterly review meetings were purposively sampled to participate in focus group discussions. Participants in the quarterly review meeting were generally those coordinating clinical teaching at their respective nursing colleges.

Subsequently, theoretical sampling was used to sample NEPI technical working group (*n* = 4), nurse educators (*n* = 12), nursing managers (*n* = 8) and nursing clinical preceptors (*n* = 8) from teaching hospitals who participated in two NEPI quarterly review meetings. Theoretical sampling shaped further data collection as the researcher pursued developing conceptual ideas and participants helped to clarify understanding (Bagnasco, Ghirotto & Sasso 2014).

#### Data collection method

Data were collected in three phases. The first phase involved analysis of reports presented at a national nursing education conference that was organised by Norwegian Church Aid (NCA) for nurse educators to share best practices in enhancing nursing education in Malawi (Table 1). During this phase, specific comments made by conference delegates were also captured and integrated into the data. Secondly, a panel discussion of eight nursing education experts and clinical practitioners who were purposively sampled on the last day of a national nursing education research conference was conducted (Table 2). The researcher posed open-ended questions guided by themes that emerged from research report analysis. The panel discussion was audio recorded after obtaining consent from the participants. Thirdly, data were collected through focus group discussions during two NEPI quarterly review meetings with nurse educators, nurse practitioners, clinical preceptors and Nurses and Midwives Council of Malawi directors guided by concepts that emerged from a panel discussion in order to fill in gaps and verify categories and concepts.

**TABLE 1:** Summary of research reports from the nursing education research conference.

Core concepts	Research report title	Key findings
Nursing context	Registered nurses’ experiences with clinical teaching environment in Malawi.	Clinical teaching and learning inadequately prepares students for practice owing to challenges of inadequate faculty support, poor clinical learning environment, poor competence among nurses and unsupportive working conditions.
	Can research improve nursing and midwifery education in Malawi – Key note.	Evidence-based practice requires that both the nurse educator and nurse practitioners engage in mutual research. Research builds on knowledge for production of nurses.
	Strategies for the implementation of clinical practice guidelines in the intensive care: a systematic review.	Practice guidelines strengthen provision of quality care to patients in intensive care unit.
Academic practice collaboration	Involvement of registered nurses in clinical teaching of nursing students in the central hospitals of Malawi.	Registered nurses possess adequate experience regarding practice. Their involvement increases the chances of narrowing a theory–practice gap that exists owing to lack of integration between what students learn in class and what is happening in practice settings.
	Assessing quality of clinical learning environment for nursing and midwifery students in northern Malawi.	The nature of the clinical learning environment has a direct impact on the achievements of the clinical learning outcomes. However, the clinical learning environment is characterised by lack of resources, poor faculty support and lack of collaboration between academia and practice in training students.
	Clinical teaching in clinical situations.	Students learn better in clinical situations that have adequate support from clinical personnel.
	Where is the grade coming from? Problems and challenges in evaluating the clinical performance of nursing students.	Evaluation of students’ clinical performance is a vital component of nursing education; it should be conducted in a manner that effectively determines students’ clinical proficiency. Consequently, students become preoccupied with building relationships with clinical nurses to obtain good grades.
Competent graduate	An investigation of stressors among Malawian nursing and midwifery students.	Clinical learning is stressful for students owing to the nature of the clinical learning environment, especially for newer students. As time passes by, students get adapted to challenges of the clinical environment.
	Patient-centred care in nursing and midwifery education.	Quality of nursing care improves when care is based on objective assessment of the patients’ needs. Nursing education emphasises evidence-based provision of nursing care.
Transformative strategies	Enhancing students’ moral competence in practice: Challenges experienced by Malawian nurse teachers.	A lesson that authoritarian learning climate may enhance critical reflection and discussion between students, teachers and nurses. Giving students more chances to reflect increases the chances of developing moral competence.
	Exploring knowledge and perceptions of tutors towards the use of problem-based learning approach (PBL) in Christian Health Association of Malawi Nursing Colleges.	Nurse educators need capacity building in teaching approaches in order to promote achievement of learning outcomes among students.
	Factors affecting clinical performance of nursing and midwifery technician students at three nursing colleges of southern Malawi.	Poor clinical learning environment that includes clinical nurses’ attitude towards students, availability of faculty support during clinical practice and lack of resources affect student performance
	Teaching and learning methodology in nurse/midwife education.	Use of various methods in teaching enhances acquisition of knowledge and skills among nurses. Clinical mentorship increases the chance for students to learn during practice.
	Knowledge and attitudes of nursing and midwifery learners and educators towards self-directed learning in Malawi.	Adequate orientation of students to teaching approaches increases students’ positive attitude towards the approaches.

**TABLE 2:** Characteristics of panel discussion experts.

Characteristics	Expertise			
	Nurse educators	Nurse practitioners	Policymakers	Regulatory
**Gender**				
Male	1	-	-	-
Female	2	3	2	1
**Age**				
< 30 years	-	-	-	-
31–40 years	1	2	-	-
> 41	2	1	2	1
**Education qualification**				
Bachelor’s	-	1	-	-
Master’s	3	2	2	1
PhD	-	-	-	-
**Years of service**				
< 5	-	-	-	-
6–15 years	1	-	-	-
> 16 years	2	3	2	1
**Publications**				
None	-	1	-	-
less than 2	1	2	1	1
More than 3	2	-	1	-

#### Data analysis

Data from the study were analysed based on grounded theory approach (Corbin & Strauss 2014) and included concept development and category saturation. Data analysis was done concurrently with data collection. Open coding involved asking questions and making comparisons to ascertain similarities and differences between concepts. More data were generated through additional questions that evolved out of the coding process. The researcher continuously re-examined, interpreted and compared data until saturation was reached. Category reduction was achieved through axial coding by linking categories to how they fitted the characteristics of a defined category. Categories were then linked together with the intention of understanding relationships among them. Categories that shared similar characteristics were merged into higher-order categories. The researcher drew logical diagrams to uncover relationships between categories (Corbin & Strauss 2014).

### Trustworthiness

Credibility of the study was achieved through data triangulation. Expert panel discussion and two focus group discussions during quarterly reviews ensured truth value of the study. Bitsch (2005) highlighted that purposive sampling of data sources increases dependability. The researchers also conducted data quality checks. Peer review of the data was also done to ensure dependability. Confirmability was ensured by tape recording and verbatim presentation of data. Emerging data were also presented to experts for verification and input into the emerging model. A detailed description of the study process including procedures and findings was done to ensure transferability (Shenton 2004).

### Ethical consideration

Ethical clearance was obtained from both the University of KwaZulu-Natal Human and Social Sciences Ethics Committee (HSS/0986/012D) and the Malawi National Health Research Ethics Committee (NHSRC 1154). Permission was sought from NEPI project manager to conduct quarterly review sessions for data collection. Informed consent was obtained and participants voluntarily took part in the study. No data were linked to individual participants to ensure anonymity. Data were treated with confidentiality.

## Results

Four core concepts emerged from an analysis of research reports that were presented at the national nursing education conference. These were nursing education context, academic–practice collaboration, competent graduate and transformative strategies (Table 1).

These core concepts formed the basis for a panel discussion and subsequent focus group discussions with nurse educators, practitioners and clinical preceptors, which resulted in the emergence of five themes.

### Theme 1: Curriculum reforms

Participants of both the panel discussion and focus group discussions highlighted the need to have curricula that respond to the healthcare users. Findings show that there is a mismatch between the type of nurses and the expectations of the service users. Some participants reported that:

‘we still train a lot of nursing midwifery technicians than registered nurses and midwives. That’s an issue of quality of nurses.’ (NE1, female, above 41 years old)

‘the country should target having registered nurses who are primary health care oriented because most users access health services at this level.’ (NP3, female, 52 years old)

Participants noted that curricula should be formulated in accordance with the actual health needs of the users.

Participants reported:

‘Our country needs nurses and midwives who are deliberately trained for the rural areas. Nurses who will serve the neglected communities.’ (PM1, female, 52 years old)

‘as a country we have not embraced interprofessional training of health workers yet. We need training programs that will foster inter-professional approach to healthcare problems and demands.’ (NE3, male, 40 years old)

Findings have shown that nursing education contributes substantially to the reduction of the healthcare workforce shortage. Some extracts from panel discussion include:

‘our nursing programs no longer produce nurses whom the healthcare delivery system could rely on to deal with the challenges among our communities.’ (PM2, female, 46 years old)

‘… there are too few nurses to serve the increasing number of patients in the health centres and health posts in remote areas.’ (NP2, female, 35 years old)

‘… the HSSP drives the agenda for all of us, be it education, practice or regulation.’ (PM1, female, 52 years old)

### Theme 2: Regulation

Findings of the study show that there is a well-established system of regulation and accreditation of nursing education in Malawi. The nurses and midwives council ensures that all nursing programmes being offered in the country are approved and accredited. Participants urged therefore that the nurses and midwives council should therefore be well conversant with the nursing care needs of the service users. Participants highlighted the importance of strategic leadership with the regulatory body in order to increase the number of competent nurses and midwives.

Participants highlighted that:

‘nurses and midwives council prescribes curricula content for nursing programs. It should be well informed of the healthcare needs in order to provide relevant nursing curricula.’ (NR1, female, 48 years old)

Participants also noted that the nurses and midwives council regulates licensure of nursing personnel.

‘nurses and midwives write licensure examination before they can practice in this country. How come then we talk about poor quality of nurses when they have been evaluated whether fit or not to practice.’ (NE2, female, 49 years old)

‘we need to rethink how we want to regulate licensure systems for our nurses and midwives.’ (NP1, female, 39 years old)

Participants highlighted that national standards of nursing education are key to ensure quality health services.

‘… various nurses and midwives council standards help to keep nurse education in Malawi in good order.’ (NR1, female, 48 years old)

‘… a joint evaluation exercise should be done with the Nurses Council to assess if programs have been formulated according to the needs of the national health system.’ (PM1, female, 52 years old)

### Theme 3: Transformative teaching

Findings of the study indicate that there is a growing number of students being enrolled in the training institutions. All participants concurred that there is overcrowding of students in the clinical area and this challenges clinical teaching of students. Some participants indicated:

‘teaching students in classroom and clinical practice is a challenge now with increased student intakes. We need to find better ways of teaching these students.’ (NE2, female, 49 years old)

‘… individualized teaching is more important now than ever. You need to ensure that each student’s learning needs are met if you are to ensure quality students.’ (NP3, female, 52 years old)

### Theme 4: Infrastructure and resources

Findings of the study show that need for more space, infrastructure and resources cannot be ignored owing to the increasing number of students. Infrastructure and resources to support transformative methods are needed at both educational and practice institutions. Participants noted that in the absence of adequate infrastructure, distance learning is becoming an option in many countries to avert challenges with space owing to increasing number of students. Participants highlighted that:

‘… massive investment is needed to provide educational institutions with adequate infrastructure and resources in order to accommodate changing number of students and need for innovative way of teaching them.’ (PM2, female, 46 years old)

‘… we have increased the nursing intakes to the extent that we cannot accommodate or trace them in the clinical sites.’ (NE2, female, 49 years old)

### Theme 5: Capacity building

Participants of the study reported that there are problems with capacity among nursing faculty in terms of clinical competences. Participants noted that over time, faculty members lose their practice competences. Findings show that there should be deliberate continuous professional development for nurse educators. Participants highlighted that improving educational capacity through nursing faculty development could be one of the several strategies to address a complex human resource problem. Participants reported that:

‘There should be deliberate effort to equip educational institutions with capacity to handle growing number of students and more importantly changing health care landscape.’ (NR1, female, 48 years old)

‘… mutual collaboration among academic and practice staff increases their chance to share expertise thereby building each other’s capacity.’ (PM1, female, 52 years old)

## Discussion of research results

Chinn and Jacobs (1987) highlighted six components of a model which include (1) goals of a model, (2) concepts, (3) definition (4) relationship statements, (5) model structure and (6) assumptions.

### Assumption of the model

The model for improving quality, quantity and relevance of nursing education was created on assumptions derived from World Health Organization framework for transforming and scaling up health professionals’ education and training (World Health Organization 2013) and synthesis of the empirical literature. Fawcett (2005) states that assumptions are principles that are accepted as true without proof. The four assumptions for this model are:

- Transforming and up-scaling of nursing education should be responsive to the health needs of societies and ensure universal coverage.
- Teaching and learning process should prepare a graduate who is ready to address the health system’s needs and demands.
- National, institutional and programme leadership commitment drives positive transformation when implemented using a collaborative and partnership approach.
- A nurse graduate who is competent, relevant to the needs of a society, builds a strong health system.

### Description of the model

The middle-range model presented in this article is aimed at guiding efforts to improve quality, quantity and relevance of nursing education in Malawi. This is a planning model and aims at providing guidance to planners to match strategies with situational determinants within the nursing education context. Planning models are developed to prevent the common practice of using one strategy to solve all problems. Although the model was developed in the Malawian context and within nursing education, it can be used in any country with similar nursing education context and in a variety of health professionals’ education.

### Substantive concepts and conceptual relationship

Chinn and Kramer (1983) stated that theorists may either use relatively associative definition (by how they are used within a theory) or relatively specific definition (by what they mean). Concepts and sub-concepts in this model have used relatively associative definition. The main concept for this model is transforming and scaling up of nursing education because it is a phenomenon of interest for the study. The other major concepts are directly linked to the main concept and include (1) nursing education context, (2) academic–practice collaboration or partnership, (3) competent graduate and (4) nursing workforce.

### Transformative and scale-up of nursing education

Transformative and scale-up of nursing education refers to a sustainable expansion and reform to increase quantity, quality and relevance of health professionals (World Health Organization 2011). Transforming and scaling up of nursing education focuses on three dimensions, namely quality, quantity and relevance. The model defines quality of nursing workforce as having adequacy of nursing qualifications to address the health needs of a specific population. Quantity is the adequacy in the number of nurses to address the health needs of a specific population. Relevance refers to achieving an appropriate skill mix and equitable distribution and availability of health professionals to the local context. Sub-concepts within this concept include curriculum reform, regulation, transformative teaching, infrastructure/resources and capacity building (Table 3).

**TABLE 3:** Showing sub concepts and supporting extracts.

Sub-concept	Supporting extracts
Curriculum reforms	‘… the national agenda should inform nursing programme developments.’ (NE3, male, 40 years old)
	‘… usually our training of our nurses is static despite the changing healthcare landscape. We need to make health system and health care education speak to each other.’ (NP2, female, 35 years old)
Regulation	‘… Nurses council should be well informed of the demands of the healthcare system because its them who sets the syllabus and expected competence of nurses and midwives.’ (NR1, female, 48 years old)
Transformative learning	‘… with growing number of students, different innovative approaches need to be suggested. For example, use of technology in teaching huge number of students.’(NE2, female,49 years old)
	‘simulation can be used to cut the demand for clinical space. Fundamental skills can be mastered right at college in the laboratory.’ (NE3, male, 40 years old)
Infrastructure	‘… infrastructure should keep pace with growing technology and number of students.’ (PM2,female 42 years old)
	‘… most colleges are failing to increase their intakes because of space. Can’t we create space so that we end the crisis.’ (NP3, female, 52 years old)
Capacity building	‘… mandatory CPD will help faculty to update themselves with current skill and competence.’ (PM2, female, 42 years old)
	‘College management teams need capacity to manage transformation.’ (PM1, female, 51 years old)
	‘… we need to get our fellow clinical staff members on board as we plan for capacity building. We can also engage each other in research, CPD….’ (NE1, female, above 41 years old)

CPD, continuous professional development.

Curriculum reform is central to transform the quality and relevance of nursing education. The recent focus on competency-based curriculum calls for identification of critical nursing activities and general competencies (Ten Cate & Scheele 2007). Competency-based learning strengthens individual-based outcomes for each student. The call for re-engineering of primary healthcare for universal access to healthcare calls for concerted effort on health promotion. Transformative education demands that curricula be responsive to the needs of the society. The poor rural communities are most often neglected and most of the healthcare policies and strategies do not address the plight of these poor populations (Goodridge et al. 2015).

Interprofessional education has been recognised worldwide as a key component in strengthening healthcare and overcoming practice challenges by teaching students the necessary skills to become part of the collaborative practice-ready health workforce (Thistlethwaite & Moran 2010). It encourages members of more than one health profession to learn interactively together for the explicit purpose of improving interprofessional collaboration and overall health of the populations. Suter et al. (2012) reported that interprofessional collaboration has been linked to a range of outcomes, including improvements in patient safety and case management, the optimal use of skills of each team member and provision of better health services. With growing task shifting within the healthcare system (Joshi et al. 2014), interprofessional education will have a positive impact on learners’ attitudes, knowledge and skills on collaboration (Sargeant, MacLeod & Murray 2011).

Regulation is one important strategic direction in the roadmap for scaling up health workforce. The function of regulation is to ensure that each individual has some level of understanding of accountability to his or her professional discipline. Regulation can be enforced at different levels. Institutions can be regulated through accreditation to ensure they meet expected standards to train nurses or for student practice in case of clinical institutions. Individuals are also regulated to ensure that they possess the relevant competences to be safe practitioners. Quality assurance in nursing is important for healthcare services users’ safety. The regional professional regulatory framework for nursing and midwifery reported that efforts to improve nursing and midwifery tend to ignore regulation (World Health Organization 2014).

With the growing number of students being recruited into nursing programmes, there should be innovations in methods of teaching and learning. In common instances, despite the growing number of students, the number of clinical practice sites have remained the same (Msiska, Smith & Fawcett 2014). Students are competing for learning opportunities and resources. Studies have shown that clinical staff usually do not have adequate time for student clinical teaching owing to duo roles. Simulation has globally been recommended as a new way to build skills among students within the college environment (Kelly et al. 2016; Shin, Park & Kim 2015).

Infrastructure and resources to support transformative methods are needed at both educational and practice institutions. In the absence of adequate infrastructure, distance learning is becoming an option in many countries to avert challenges with space owing to the increasing number of students (Graber 2015).

Capacity building as a sub-concept spurs around three dimensions. Firstly, studies have reported that the productive capacity of training institutions in many sub-Saharan countries is low. Training institutions cannot recruit adequate number of students. Appiagyei et al. (2014) highlighted several reasons including shortage of educators, overcrowding in the clinical area and lack of teaching and learning resources and infrastructure. Improving infrastructure will allow colleges to take in more students. Secondly, problems with capacity among nursing faculty in terms of competences have been reported for decades (Roberts 2015: Solum et al. 2015).

Mandatory continuous professional development is needed for faculty (Breakey et al. 2015). Many studies have reported that faculty members lose their practice competence over time as they work in training institutions. Improving educational capacity through nursing faculty development has been proposed as one of the several strategies to address a complex human resource problem (Middleton et al. 2014).

### Nursing education context

Nursing education context in this model refers to the present situation in the country serving as an antecedent for developing a model that will enhance nursing education in the country in terms of quantity, quality and relevance.

Malawi like many other sub-Saharan African countries still reports poor health indicators. Several factors have been attributed to the situation including inadequate financing of the health systems (Lu et al. 2010), severe shortage of health workforce, especially nurses (Bradley et al. 2015), high illiteracy level and lack of competent health service providers. There is a disjoint between competence needs and population’s health needs (Frenk et al. 2010). In a country where the majority of the population reside in hard to reach disadvantaged areas, a community-based and primary healthcare approach would be relevant (Stigler et al. 2016). The model is built on the assumption that building an efficient comprehensive nursing education system will strengthen health service delivery (Middleton et al. 2014).

The structure of the model shows that the need for transformation should overlap within all levels of strategic implementation. Transformation needs to take place at national, institutional and programme levels guided by policy and frameworks that define national agenda for health. The national health needs are central and provide policy direction.

### Political agenda and strategic direction

Political agenda and strategic direction refer to governments’ and nursing education stakeholders’ plans with regard to nursing education improvement, expansion or reforms. Malawi is a United Nations member state and it abides by conversations and protocols set by United Nations through its agencies to improve the well-being of humanity. The political agenda and strategic direction is propagated down the system from national, institutional to programme levels. The National Health Sector Strategic Plan 2011–2016 highlighted the importance of transforming nursing education. The main goal of the Government of Malawi is to have the highest possible level of health and quality of life for all Malawians. Institutions and programmes have to set their agenda to be responsive to the national goal. Lu et al. (2010) noted that political agenda and strategic direction should be pursued with equal commitment for financial input for institutions to achieve set objectives.

National health policies, strategies and plans play an essential role in defining a country’s vision, priorities, budgetary decisions and course of action for improving and maintaining the health of its people (Dahlgren & Whitehead 2016). Equally important within the political agenda and strategic direction concept is the Nurses and Midwives Council of Malawi (NMCM). As a regulatory body for nurses and midwives, NMCM sets standards for training, education and practice. The council sets expected levels of competence for nursing graduates for safe practice (Seboni et al. 2013). The council develops appropriate curricula for each cadre and hence should also foster appropriate implementation of such programme through programme evaluation (WHO/AFRO 2008).

**FIGURE 1:**
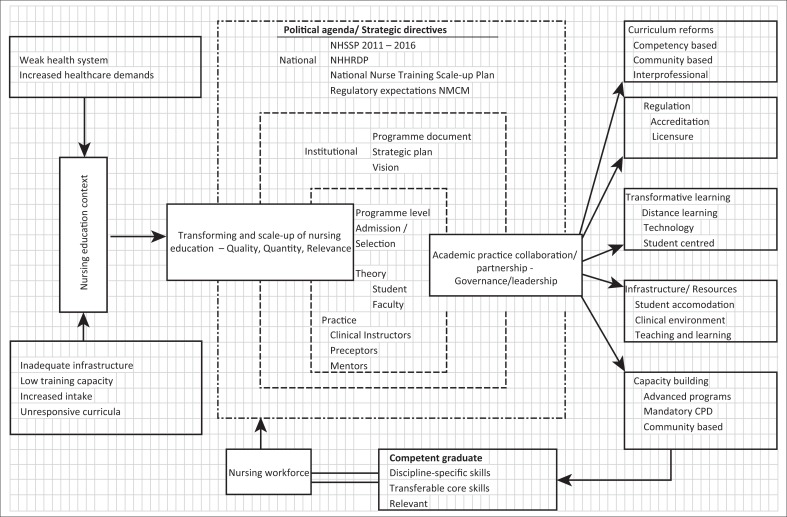
A middle-range model of improving quality, quantity and relevance of nursing education in Malawi.

Figure 1 depicts that for national goals to be successfully achieved, collaboration has to take place at national, institutional and programme levels. There should be mutual and shared goal and resource sharing. The higher level organisation’s goals feed plans of the lower level organisation.

### Academic–practice collaboration or partnership

Partnership refers to a deliberate arrangement between parties to cooperate to advance mutual interest. In this model, academic practice partnership is defined as a well-defined strategic relationship between educational and clinical practice settings in the area of practice, education and research. Student nurses generally rotate between blocks of theory and clinical practice. The model advocates for formal structured relationships towards achieving the country’s health sector objective of health for all. The model is also built on the assumption that a health system consists of all organisations, people and actions whose primary interest is to promote health, in which collaboration is critical (World Health Organization 2007).

The complexity of the healthcare environment demands collaboration between academia and practice setting in preparing an effective healthcare workforce (Frenk et al. 2010). Academic practice partnership is an important mechanism to strengthen nursing education, practice and research and help nurses become well positioned to lead change and advance health. Missal et al. (2010) point out that academic practice partnership provides leadership, mentorship and support in collaborative practice, thereby facilitating reduction of theory practice gap among students and nursing staff. Academic–practice partnerships enhance learning culture as both academics and practitioners take upon themselves to ensure well-trained and socialised graduates (Schinka & Raia 2013). There are high chances of success when strategies being put in place to transform nursing education for effective health systems are implemented within this formal collaboration.

Figure 1 shows that all strategies should be implemented through a collaborative approach.

### Competent graduate

Competency refers to the expected level of performance integrating knowledge, skills and attitudes. In the model, a competent graduate is one who demonstrates the ability to perform based on this expected level after training. Kajander-Unkuri et al. (2013) identified eight competence areas of importance for nursing students, including professional and ethical values and practice; nursing skills and intervention; communication and interpersonal skills; knowledge and cognitive ability; assessment and improving quality in nursing; professional development; leadership, management and teamwork; and research utilisation.

The nature of the curricula and amount of time students engage in putting theory into practice determine competence levels of students (Rahmati et al. 2015). Community-based education has gained more preference in most countries (Horwood et al. 2015). World Health Organization (1993) reported that the conventional approach focused on hospital-based, curative-focused teaching which relies on sophisticated technology that is not available where the majority of the people live. The approach also results in many graduate nurses refusing to work in rural, underprivileged areas after graduation. Reforms in recruitment could help the situation.

The poor rural communities are most often neglected (De Kock & Pillay 2016). Government should introduce ways that are inclusive and support the rural communities in improving their health and livelihoods. Students from the rural area could be supported and bonded to work back in the rural area after completion. The graduate needs to be supported to effectively adapt to the demands of the health system as early as possible. Transition to practice policies could range from skill mix and location-based support. The model highlights transition to practice with single dotted lines between competent graduates and the nursing workforce. With an effective transition of graduates, the health system will be sustainable with adequate leadership to drive it forward.

### Nursing workforce

Nursing workforce in this model refers to a system of qualified and licensed nurses that form part of the healthcare workforce. Malawi has nurse midwife technician and registered nurse cadres. Enrolled nurses are trained to a minimum of college diploma while registered nurses are trained from the level of university diploma or higher.

Healthcare workforce is a key building block of health systems (Essack 2012). It affects access to care as well as the quality and cost of effective delivery of services (Anand & Bärnighausen 2012). A well-performing health workforce is responsive, fair and efficient to achieve the best health outcomes. There should be sufficient number and mix of staff, which is fairly distributed (Fulton et al. 2011). The World Health Organization’s 2007 Framework for Action for strengthening health systems in developing countries identified quality as one of the key drivers of improved health outcomes and greater efficiency in health service delivery. Nurse midwife technicians are trained to work in the rural area. Most of the registered nurses are posted to tertiary hospitals other than where they are needed most. However, International Council of Nurses (2012) identified registered nurses as a professional group that is crucial for providing high-quality and safe care. Muula (2006) reported that even at the legislation level, members of parliament strongly advocate that the lower cadre be trained to ensure that they do not leave the healthcare system for developed countries.

### Evaluation of model

The model was evaluated in accordance with the criteria proposed by Fawcett (2005) for model evaluation, which addressed questions regarding its significance, internal consistency, parsimony, testability, empirical adequacy and pragmatic adequacy. A self-reflective questionnaire to evaluate the model was formulated under the guidance of an expert supervisor. Significance focuses on the context and justifies the importance of the theory. Internal consistency was ensured in this study by consistent definition of concepts that gave semantic clarity and semantic consistency. Parsimony was achieved by giving clear and concise statements of concepts. A series of qualitative studies that were done on the faculty’s perceptions and experiences on implementation of transformative nursing education culminated in the testability and empirical adequacy of the model. The similarity of numerous frameworks to guide transformation and scale-up of nursing education evaluates the pragmatic adequacy of the theory.

## Limitations of the study

The study was conducted to guide implementation and evaluation of transformative and scale-up of nursing education in the local context of Malawi. Guidelines provided by key stakeholders like the World Health Organization remain relevant to the implementation of the model.

## Recommendations

For nursing education – The model has been developed to guide implementation and evaluation of strategies to improve the quality, quantity and relevance of nursing education.

For research – More research is needed to explore the impact of specific strategies using the model through comparative studies between Malawi and other countries with a similar context.

## Conclusions

The design and assumptions of the model were discussed followed by a description of concepts and relationship among concepts. Nursing education transformation and scale-up should take place within a collaborative environment that fosters mutual strategic and operational planning within the framework of willing and committed leadership. Leadership cuts across approaches at national, institutional and programme levels among all nursing education stakeholders.
